# Some Reflections on Intracytoplasmic Morphologically Selected Sperm Injection

**Published:** 2014-07-08

**Authors:** Thomas Ebner, Omar Shebl, Peter Oppelt, Richard Bernhard Mayer

**Affiliations:** Department of Gynecological Endocrinology and Kinderwunsch Zentrum, Landes-Frauen-and Kinderklinik and Faculty of Medicine, Johannes Kepler University, Linz, Upper Austria, Austria

**Keywords:** Chromatin, DNA, ICSI, Sperm Head, Vacuole

## Abstract

Although intracytoplasmic sperm injection (ICSI) allows proper fertilization in most cases of male sub fertility, it is one of the most unphysiological techniques in assisted reproductive technologies (ART). Thus, over the last decade, researchers have tried to improve sperm observation with higher-resolution microscopy techniques such as the intracytoplasmic morphologically selected sperm injection (IMSI) technique. In order to identify literatures for this review, the PubMed database was searched from 2000 onwards using the terms IMSI, motile sperm organelle morphology examination (MSOME) and sperm vacuole. Approximately 10 years after the introduction of the MSOME and IMSI procedures, several questions related to the prevalence, origin, location, and clinical consequences of sperm vacuoles have not yet been clarified. It seems that IMSI as a routine application is not state of the art and the only confirmed indications for IMSI are recurrent implantation failure following ICSI and severe male factor.

## Introduction

Despite the fact that intracytoplasmic sperm injection (ICSI) allows successful fertilization in virtually all cases of male subfertility it is evident that ICSI is one of the most unphysiological methods of assisted reproductive technologies (ART). It is not only that major processes of *in vivo* fertilization are circumvented, but ICSI also changes the physiological conditions.

Firstly, incorporation of the unreacted acrosome into the oocyte during ICSI could be potentially hazardous to embryo development since there is an increased risk to the formation of vacuoles in the female gamete due to acrosomal enzymes (1, 2). Additionally, in a mouse model Ca^2+^ responses significantly differed in terms of duration, frequency and amplitude between IVF and ICSI (3). Last but not least, a dramatic change in gene expression has been reported in ICSI as compared to the *in vivo* situation in the mouse. Mostly, genes related to cell function and development were found to be up- or down regulated (4).

Apart from these physiological abnormalities clinical embryologists applying routine sperm selection criteria at ×400 magnification take a higher risk of selecting male gametes defective incentrosomal integrity (5, 6), genetical constitution (7), phospholipase C zeta content (8), protamine ratio (9) and/or DNA-methylation (10).

To avoid this scenario or at least to reduce the potential effects of ICSI using suboptimal sperm, every effort must be taken to deselect abnormal and to accumulate good prognosis spermatozoa (e.g., by applying particular sperm processing methods). This quest for optimized male gamete selection has been summarized as physiological ICSI (11, 12).

In principle, four different parameters of sperm morphology are conceivable that would allow to identification of physiological sperms: i. DNAintegrity (13, 14), ii. Birefringence (15, 16), iii. Maturity (17) and iv. High-resolution morphology (18, 19).

The problem with DNA-strand break testing is that it is almost impossible to do on living sperm cells (20, 21). To put it differently, most current tests irreversibly damage the sperm analyzed, thus, it will not usable in ICSI. Polarization microscopy for evaluating the highly ordered filaments of the head and neck region requires special changes in the setup of polarization microscopes which are not manageable at all centers. Lastly, it seems that checking hyaluronic receptor bonding capacity as a major characteristic of sperm maturity is strongly dependent on laboratory setup such as temperature control.

Therefore, it is hardly surprising that ICSI at very high magnification (at least ×6000) reflects the majority of research on this matter in literature.

### Intracytoplasmic morphologically selected sperm injection (IMSI)

To overcome the limits of conventional microscopy, Bartoovet et al. developed a method of unstained, real-time, high magnification examination of spermatozoa. This particular high magnification scoring of spermatozoa is known under the term motile sperm organelle morphology examination (MSOME) and exclusively deals with the presence, size, number, and location of vacuoles. Per definition, MSOME criteria consider nuclear chromatin content to be abnormal if the sperm head contains one or more vacuoles (diameter of 0.78 ± 0.18 ìm) occupying more than 4% of the normal nuclear area (22-24). It turned out that MSOME represents reliable criteria as sequential analyses of sperm samples from the same patients produced similar results in terms of normal morphology and presence of large vacuoles (25).

For the ease of adequate scoring, the MSOME criteria have been used by several working groups (26) to subgroup sperms into different classes which helped to simplify statistics. In short, grade I spermatozoa exclusively consisted of sperms showing normal sperm head and absence of vacuoles, thus, representing the optimal type. Grade II gametes were made up of sperms showing a maximum of two small vacuoles. Grade III spermatozoa were characterized by the presence of more than two small vacuoles or at least one large vacuole. The worst grade IV showed large vacuoles in consumption with head shape problems and other abnormalities. Others used a similar model to classify abnormal spermatozoa by their degree of vacuolization (27). Cassuto et al. (28) introduced the so-called HAVBIC criteria based on the detailed analysis of head, acrosome, vacuoles, basis of the sperm head, insertion which is the axial position of the tail, and the presence of a cytoplasmic droplet.

However, MSOME examination usually is performed utilizing an inverted light microscope equipped with high-power Nomarski optic enhanced by digital imaging to achieve a magnification of up to ×6300.

Injection of spermatozoa selected by the above mentioned MSOME criteria culminated in a modified ICSI technique called IMSI (29). Its introduction in the field of ART definitely facilitated the observation of live human spermatozoa, particularly by showing sperm vacuoles not necessarily seen at lower magnification, prior to injection in the oocyte. It soon turned out that precise morphological integrity of the human sperm nucleus is an important parameter associated with pregnancy rate (29, 30). In detail, the inventors of the IMSI technique showed a significantly increased pregnancy rate in IMSI (66%) as compared to routine ICSI (30%). The associated implantation rate was even the 3-fold (9.5 vs. 27.9%). In case that no optimal sperm was available for IMSI an increase in abortion rate from 10 to 57% was described (30).

Of course it has to be kept in mind that early research in this field has more or less been performed by one working group, thus, a potential bias in their interpretation of the data cannot be excluded (e.g., an abortion rate as high as 57% is incredibly high).

### Where do vacuoles come from?

IMSI is a rather time-consuming procedure since selecting enough morphologically normal spermatozoa for injection according to the above mentioned MSOME criteria may take up to 2 hours (31). It has been suspected that this prolonged process of searching for spermatozoa at high magnification might damage the male gametes since it had been observed that after 2 hours on the micro scope’s heated stage sperm nucleus vacuolization showed a significant increase (32).

Recently, Neyer et al. (33) nicely highlighted that sperm head vacuoles are rather not affected by *in vitro* conditions as analyzed by a system of microchannels. This sperm-microcapture area allowed observation of the same living human spermatozoon over a period of 24 hours. In a series of experiments it was demonstrated that neither temperature nor oxidative stress led to the formation of de novo vacuoles. On the other hand, induction of the acrosome reaction using the ionophore A23587 did not lead to disappearance of vacuoles (33). Recently, it was shown that freezing-thawing procedures also did not influence the relative vacuole area in the anterior, medial or basal part of the sperm head (34).

Based on these data it appears evident that vacuoles are structures being pre-existing and their number cannot be altered by *in vivo* conditions. This is in line with data from a Japanese group (35) who found that the distribution of vacuoles in ejaculated (98.3%), epididymal (87.5%), and testicular sperm (87.5%) and in spermatids (33.7%) was directly correlated to maturational stage of the germ cell. In other words, the closer a sperm gets to ejaculation, the higher is its risk to bear one or more vacuoles. It seems that particularly the time of acrosome reaction is critical. Kacem et al. (36) reported that of all acrosome-reacted sperm analyzed 70.9% were free of vacuoles, whereas in those sperms showing incomplete acrosome reaction or an intact acrosome at all the corresponding percentage was only 39.3%. This strongly suggests that IMSI selects acrosome-reacted spermatozoa. And it is in line with Montjean et al. (37) who found that sperm vacuoles are associated with acrosomal and capacitation status, that is to say, they appear to be a reflection of normal sperm physiology. However, at least in globozoospermia a non-acrosomal origin of vacuoles is discussed (38).

### Where are vacuoles located?

It seems that the actual location of all types of vacuoles is random. Information on the actually preferred site of vacuoles is scarce. Tanaka and co-workers (35) calculated that >60% of all vacuoles are found in the acrosomal region which approximately represents the anterior two thirds of the sperm head. Dividing the sperm head area into three sections, 40.9% of normal-shaped sperm had vacuoles in the tip, 74.9% in the middle region and only 4.3% in the posterior area. Irrespective of their location, a total of 92.8% of vacuoles were small and only 4.6% were large (39). In a paper of Perdrix et al. (40) 38.0 ± 5.10% of motile spermatozoa obtained after gradient density centrifugation showed a large vacuole occupying >13% of the total sperm head area. Again these vacuoles could mainly be detected in the anterior (45.7 ± 2.9%) and median sperm head (46.1 ± 3.0%). This statement is somewhat in contrast to the transmission electron microscopy (TEM) data from the same study which indicated that the large vacuoles were exclusively present in the nucleus. This could be due to an incomplete reconstruction of all TEM sections since a maximum of 1.5μm (70 nm sections ×20 cuts) of the sperm diameter was depicted. The total dimension in the posterior region would be around 3μm (41). Alternatively, this is a problem with all the vacuolization data presented so far, interpretation of the images was based on the assumption that vacuoles in sperm have the same background as vacuoles found in oocytes, e.g., being cavities within the cytoplasm (42).

More recent work applying sophisticated technologies such as three-dimensional deconvolution and atomic force microscopy clearly showed that in "all vacuolated spermatozoa the acrosome was intact, the plasma membrane was sunken but intact and the large vacuole was identified as an abnormal, thumbprint-like nuclear concavity covered by acrosomal and plasmic membranes" (41, 43).

### Clinical consequences of vacuoles

There is a relative heterogeneity between semen samples, so that the frequency by which good spermatozoa can be selected varies greatly among patients. De Vos et al. (44) demonstrated that only 5/350 (1.4%) male factor patients have to face the problem that no sperms better than grade III (26) are available for injection. This automatically means that at least in some poor prognosis patients only suboptimal sperm can be injected.

Things get clearer if the attention is drawn to a model in which actively damaged spermatozoa were used (45-48). This approach exclusively ended up with normal fertilization and cleavage, while blastomere number on day 3 (48) and blastocyst development (45, 46) were very much related to the degree of damage. Although the above cited colleagues actively damaged the DNA of the spermatozoa, rather than injecting vacuolized sperms, there is evidence that a possible negative paternal effect on *in vitro* development will not develop before embryonic genome expression. This would explain why no studies are available indicating a correlation between sperm vacuolization and fertilization rate (26, 44, 49, 50). In addition, no advantage of IMSI over ICSI was seen in terms of day 2 morphology (51).

Vanderzwalmen et al. (26) reported identical numbers of zygotes and developmental rates up to day 3 between IMSI and ICSI irrespective of the fact whether grade I or IV sperms were injected. However, blastocyst formation was found to be superior in IMSI. The worse the grade of the injected spermatozoon was, the lower was blastulation (56.3, 61.4, 5.1, 0%). Blastulation was also significantly increased in IMSI as compared to ICSI in the paper of Knez et al. (49). Interestingly, time lapse analysis revealed that blastocysts from grade I spermatozoa required the shortest mean time for all developmental events as compared with blastocysts from spermatozoa of other classes showing vacuoles (52). In some developmental phases there was a 10h-lag between embryos from grade I and IV which led to the observation that only early blastocyst could be seen on day 5 if grade IV spermatozoa were used for ICSI.

The higher availability of blastocysts for transfer could be one reason for the observed increase in pregnancy rate. Apart from the manuscripts discussed above, numerous authors have suggested an advantage of IMSI over ICSI in different patient cohorts (53). In a group of 125 couples (54), IMSI improved clinical outcomes (implantation, pregnancy) without affecting biological outcomes (fertilization and cleavage rates, embryo morphology).

Since live-birth rate represents the ultimate success of ART, IMSI studies with this parameter as primary outcome are of particular interest. Recently, Greco et al. (50) compared IMSI with good prognosis (class I and II) and bad prognosis sperm (class II and IV). In this publication the "late" outcomes like implantation (23.1 vs. 7.0%), clinical pregnancy (41.7 vs. 17.1%), and live-birth rate (36.7 vs. 14.3%) were statistically significantly higher in the patients with better MSOME quality as compared to the bad prognosis counterpart. Others (55-57) confirmed a significant trend towards a higher live-birth rate with IMSI (21-38 vs. 12-20%).

There is evidence that IMSI favors injection of genetically normal spermatozoa (58). In this article major malformations were significantly reduced in IMSI newborns versus ICSI ones (1.3 vs. 3.8%). This could be associated with an increased incidence for sex chromosome aneuploidy and chaotic embryos in ICSI but not in IMSI cycles (59). A possible impact of IMSI on gender of the offspring is still under discussion (60, 61).

### How do vacuoles affect performance of the sperm?

With the above mentioned data in mind, it seems to be very likely that IMSI based on MSOME (29), HAVBIC (28) or Vanderzwalmen criteria (26) deselects spermatozoa of reduced potential.

Indeed, Garolla et al. (61) analyzed mitochondrial function and aneuploidy rate in preselected sperms and claimed better results in these MSOME sperms as compared to an unselected counterpart. The aneuploidy part of the study was later confirmed by another working group (40). However, it is evident that IMSI does not decrease the aneuploidy rate in patients who are heterozygous for reciprocal translocations (62). In addition, sperm maturity as assessed as hyaluronic acid binding and sperm nucleus normalcy rate seem to go hand in hand (63).

Beyond that it was emphasized that strand breaks occur more frequently in spermatozoa with large nuclear vacuoles (64). Numerous papers jumped on the bandwagon and stated that DNA integrity and sperm head vacuolization are negatively correlated (65-68).

More recently, a growing body of colleagues did not support the said correlation between the presence of sperm head vacuoles and other sperm parameters (69-71). One explanation for this divergence could be the fact that the vast majority of studies analyzed DNA fragmentation and sperm vacuolization in two different populations of the same ejaculate not allowing for proper prediction. As far as known, there are only few studies fulfilling the requirement of performing different methods on the same spermatozoon. This is the only approach to ensure insights on whether a vacuolized spermatozoon reflects other abnormalities (DNA integrity, maturity, chromatin status, aneuploidy) as well.

Those that did fulfill this prerequisite did not find any correlation between vacuolization and strand breaks or aneuploidy (39, 41). The comparable incidence of structural DNA breaks in sperms with or without a large vacuole was 9.1 and 4.4%, respectively (41). These results strongly indicate that DNA damage is not responsible for or associated with sperm head vacuoles.

Since it is clear that small as well as large vacuoles are in fact indentations of the sperm head (41, 43) chromatin displacement and failure in chromatin condensation is a threatening consequence which in turn could cause epigenetic effects. This unique finding places special emphasis on those publications that noted an association between vacuoles and chromatin status (40, 41, 61).

### Prospective comparison between ICSI and IMSI

Certainly there was a hype about IMSI in the past. This method was chosen to overcome problems related to oocyte quality (72), maternal age (73), and complete developmental arrest (49). In particular at the beginning of the IMSI era, it seemed that IMSI was the solution for all problems; however, the more information was gathered on the nature of vacuoles and their possible impact on sperm physiology the less publications could be placed.

This declining interest in IMSI is reflected in two meta-analyses. The first one of Souza Setti et al. (74) postulated higher rates of implantation [odds ratio (OR: 2.72)] and pregnancy rates (OR: 3.12), but a lower miscarriage rate (OR: 0.42) in IMSI cases as compared with ICSI cases. The problem with this meta-analysis, however, is that it was only based on a limited number of three prospective trials (29-31).

A more recent meta-analysis, a Cochrane Review (75), based on nine prospective randomized controlled trials did not support previous results. In detail, as many as 1002 IMSI and 1012 ICSI cycles were compared. Neither live birth [risk ratio (RR: 1.14) ] nor abortion rate (RR: 0.82) could be altered by the IMSI technique. None of the studies included reported congenital abnormalities. Incredibly, the fact that clinical pregnancy rate was significantly increased (RR: 1.29) did not keep the authors from downgrading the quality of this evidence because of imprecision, inconsistency, and strong indication of publication bias.

A recent sibling-oocyte study on more than 3,000 oocytes compared conventional ICSI with a sperm selection method using higher magnification. No differences in oocyte fertilization rate or in embryo quality were observed. Also, the clinical pregnancy rate and the implantation rate per embryo transferred were similar for IMSI-only and ICSI-only transfers; thus, these data do not support any benefit of IMSI in a non-selected population with fresh ejaculated sperm containing ≥1 million/ ml (44). IMSI in another prospective randomized trial (76) did not show a significant improvement in the clinical outcome compared with ICSI although the authors found trends for higher implantation (28.9 vs. 19.5%), clinical pregnancy (54.0 vs. 44.4%) and live birth rates (43.7 vs. 38.3%) in the IMSI group.

## Conclusion

With no clear consensus regarding the effect of IMSI on implantation or pregnancy rates, IMSI is most likely a procedure in ART to be reserved for specific cases (77). Even if IMSI is chosen as the method of choice, in the vast majority of cases several sperms of good morphology (e.g., grade I and II) will be collected and used for injection. Thus, the question arises when to apply IMSI at all.

There is evidence that inflationary routine application of MSOME criteria in order to select the best spermatozoon is not required (78). In 255 couples attempting their first ART attempt for male infertility a prospective randomized trial was performed to compare the clinical outcomes of IMSI and ICSI and to evaluate the influence of sperm characteristics on these outcomes. It turned out that the results of IMSI were similar to the ICSI ones whatever the degree of sperm DNA fragmentation, nuclear immaturity and sperm morphology. These results show that IMSI instead of ICSI has no advantage in the first ART attempts (79), thus supporting the results of Balaban et al. (76) and De Vos et al. (44).

However, in a subsequent cycle to a failed ICSI, pregnancy and delivery rates were significantly higher for patients deciding to switch to the IMSI technique as compared with patients staying with ICSI (80). In an additional prospective study in which couples acted as their own controls, 75 infertile couples were offered IMSI after at least two previous treatment failures (81). IMSI seemed to give better embryo quality and more blastocysts, which allow more embryo transfers at the blastocyst stage and consequently an increased pregnancy rate.

In contrast to a recent review of Boitrelle et al. (82), El Khattabi et al. (57) reported identical live birth rates in IMSI (21%) and ICSI (22%) in cases with repeated implantation failure. Yet, another subgroup with male factor infertility benefitted from the IMSI technique since there was a significant improvement in live birth rate (38 vs. 20%). These findings are in line with those reported by others (52, 76).

In cases of severe male factor infertility, such as patients with high sperm DNA fragmentation rates, selection of normal spermatozoa with a vacuole-free head using IMSI yields the greatest likelihood of obtaining pregnancies. Successful pregnancy and healthy childbirth were also obtained in a case of total globozoospermia after MSOME/IMSI without assisted oocyte activation (83). Despite conflicting published results teratozoospermia is the preferential indication for MSOME and IMSI, but this has to be confirmed in future studies.
